# Plasma-Derived Exosomal SncRNA as a Promising Diagnostic Biomarker for Early Detection of HBV-Related Acute-on-Chronic Liver Failure

**DOI:** 10.3389/fcimb.2022.923300

**Published:** 2022-07-07

**Authors:** Wenli Xu, Mingxue Yu, Yuankai Wu, Yusheng Jie, Xiangyong Li, Xinxin Zeng, Fangji Yang, Yutian Chong

**Affiliations:** The Department of Infectious Diseases, The Third Affiliated Hospital of Sun Yat-sen University, Guangzhou, China

**Keywords:** Acute-on-chronic liver failure, SncRNA, Exosomes, Chronic hepatitis B, Diagnosis

## Abstract

**Objectives:**

The small noncoding RNAs (sncRNAs) including microRNAs and the noncanonical sncRNAs [i.e., tRNA-derived small RNAs (tsRNAs) and rRNA-derived small RNAs (rsRNAs)] are a vital class of gene regulators in response to a variety of diseases. We focus on an sncRNA signature enriched in hepatitis B virus (HBV)-related acute-on-chronic liver failure (ACLF) to develop a plasma exosome-based noninvasive biomarker for human ACLF.

**Methods:**

In this work, sncRNAs related to HBV-ACLF were identified by small RNA sequencing (RNA-seq) in plasma exosomes collected from 3 normal subjects, 4 chronic hepatitis B (CHB) patients with flare, and 6 HBV-ACLF patients in the discovery cohort. Thereafter, the differentially expressed sncRNAs were further verified in a validation cohort (n = 313) using the newly developed molecular signature incorporating different mi/ts/rsRNAs (named as MTR-RNAs) through qRT-PCR assays. Subsequently, using the least absolute shrinkage and selection operator (LASSO) logistic regression (LR) model analysis, we developed an MTR-RNA classifier for early detection of ACLF.

**Results:**

The identified sncRNAs (hsa-miR-23b-3p, hsa-miR-223-3p, hsa-miR-339-5p, tsRNA-20, tsRNA-46, and rsRNA-249) were specifically differentially expressed in plasma exosomes of HBV-ACLF. The MTR-RNA signature (AUC = 0.787) containing the above sncRNAs distinguished HBV-ACLF cases among normal subjects with 71.67% specificity and 74.29% sensitivity, CHB patients with flare (AUC = 0.694, 85.71% sensitivity/59.5% specificity), and patients with CHB/cirrhosis (AUC = 0.785, 57.14% sensitivity/94.59% specificity). Notably, it revealed 100% specificity/94.80% sensitivity in detecting patients or normal people.

**Conclusions:**

Our as-constructed plasma-derived exosomal sncRNA signature can serve as a reliable biomarker for ACLF detection and also be adopted to be the pre−triage biomarker for selecting cases that can gain benefits from adjuvant treatment.

## Introduction

Acute-on-chronic liver failure (ACLF) accounts for a kind of acute liver function deterioration, which also involves substantial liver damages. It has a great short-term death rate as high as 50%–90% (Wu et al., 2018) and occurs frequently in patients infected with hepatitis B virus (HBV) in African and Asia-Pacific areas (Trépo et al., 2014; Bernal et al., 2015; Chen et al., 2015). HBV-ACLF may be found during diverse chronic HBV infection stages due to immunosuppression-induced or spontaneous reactivation or chronic hepatitis acute deterioration (Oketani et al., 2014). Acute exacerbation refers to the severe liver injury occurring in chronic hepatitis B (CHB) patients during a hepatitis flare (Kumar et al., 2009; Shouval, 2014), and up to 8% of patients with hepatitis flares will develop decompensation (Zhao et al., 2018). There is growing evidence suggesting that CHB patients experiencing severe acute exacerbation (SAE) may be associated with a risk of ACLF (Zhao et al., 2018). HBV-ACLF is associated with poor short-term prognostic outcome, unless emergency liver transplantation can be conducted. Only a few cases can receive liver transplantation because it is expensive and the liver source is lacking. Under such background, identifying ACLF risk in chronic HBV infection cases with SAE early is vital to take effective treatment and aggressive management in advance to lower the incidence of ACLF.

However, universal and reliable predictive biomarkers of HBV-associated ACLF are lacking. Furthermore, numerous challenging problems remain to be solved. Consequently, it is important to discover the creditable and efficient ways to monitor high-risk individuals and to detect ACLF early, so as to improve patient prognosis.

Liquid biopsy is a revolutionary strategy in a variety of diseases, including chronic or acute-on-chronic disease, diagnosing or predicting tumor survival, and it is applied for analyzing tumor cells or inflammation or their generated products *via* biofluids such as urine and blood (Li et al., 2021). Exosomes represent the extracellular vesicle (EV) subset, which are 30–150 nm in diameter and have a lipid bilayer membrane (Dao et al., 2022). *Via* delivering specific cargos, including protein, DNA, and RNA [messenger RNA (mRNAs) and noncoding RNAs (ncRNAs) including microRNAs (miRNAs), circular RNAs (circRNAs), or long noncoding (lncRNAs)], exosomes make important effects on regulating intercellular crosstalk under physiological or pathological conditions (Qu et al., 2016; Li and Xu, 2019; Wang et al., 2019). Exosomes have been increasingly suggested to be related to cancer occurrence, metastasis, drug resistance, and immunomodulation through modulating extracellular communication (Valadi et al., 2007; Kahlert et al., 2014; Kalluri, 2016; Li and Xu, 2019; Hoshino et al., 2020). Recently, experiments *in vitro* and *in vivo* have demonstrated the important effect of exosomes on hepatocellular carcinoma (HCC) occurrence, diagnosis, progression, and treatment (Abudoureyimu et al., 2019). Therefore, as the novel liquid biopsy type, exosome cargos may be used to diagnose and/or predict the prognosis of ACLF.

Existing studies regarding nucleic acid biomarkers mostly focus on miRNAs, while the novel findings about noncanonical small noncoding RNAs (sncRNAs), including tRNA-derived small RNAs (tsRNAs) and rRNA-derived small RNAs (rsRNAs) (Shi et al., 2018), and their emerging roles in the dysregulation, metabolism, and diagnosis of ACLF disease are rarely reported. tsRNA, which is generated from precursor or mature tRNA, can be divided into tRNA-derived fragments (tRFs) (Kumar et al., 2016) and tRNA halves (tiRNAs) (Giege, 2008). ts/rsRNAs are extensively identified within diverse species (Shi et al., 2018; Shi et al., 2021). tsRNAs have aroused wide attention for their biological effects recently, and they are related to different human disorders (Balatti et al., 2017; Zhu et al., 2019; Farina et al., 2020; Gu et al., 2020; Su et al., 2020; Huang et al., 2021). However, rsRNAs show a sensitive response to pathophysiological conditions (Zhang et al., 2018), and the combination of miRNA, tsRNA, and rsRNA in HBV-ACLF has not been reported.

Herein, we performed plasma-derived exosomal small RNA sequencing (RNA-seq) to analyze the all-time dynamic landscapes for miRNAs, tsRNAs, and rsRNAs in the HBV-ACLF progress. Then, using the LASSO logistic regression (LR) model analysis, we established a signature that included differentially expressed miRNAs, tsRNAs, and rsRNAs at diverse HBV infection stages to assess the role of sncRNAs in HBV-ACLF early diagnosis. The results of this study may have certain clinical application prospects in the future.

## Materials and Methods

### Study Subjects

In total, 326 participants admitted to the Third Affiliated Hospital of Sun Yat-sen University were recruited for the present study between December 2020 and July 2021. The discovery cohort (n = 13) included healthy controls (n = 3), CHB patients with flare (n = 4), and HBV-ACLF patients (n = 6), whereas the validation cohort (n = 313) included healthy controls (n = 60), patients with HBV-associated cirrhosis or CHB patients (n = 69), CHB patients with flare (n = 69), and HBV-ACLF patients (n = 115). The enrolled cases were hospitalized due to an over 6-month history of hepatitis B surface antigen (HBsAg) positiveness. CHB cases with flare were included according to the criteria made by Tsubota et al. (2005) and Wong et al. (2011), including international normalized ratio (INR) <1.5 and alanine transaminase (ALT) ≥10 upper limit of normal (ULN). Meanwhile, patients with CHB or HBV-related cirrhosis were included following the criteria of INR <1.5, total bilirubin (Tbil) <2 ULN, ALT <5 ULN, and with/without cirrhotic changes on images. The diagnosis of HBV-ACLF was made with reference to 2014 APASL diagnostic guidelines (INR >1.5, Tbil >5 ULN, with hepatic encephalopathy or ascites in the past 2 weeks). Additionally, the current work also recruited normal subjects at the Staff Physical Examination Center of the Third Affiliated Hospital of Sun Yat-sen University. **Table 1** presents the demographics and clinical information of all participants.

**Table 1:** Demographics and clinicopathologic characteristics of patients.

Characteristics	Healthy control (referring to N)	HBV others (CHB/Cir) (referring to C)	HBV-flare (referring to H)	HBV-ACLF (referring to L)	*P*-value
Case number	60	69	69	115	
Age (years)	40.33 ± 10.92	47.04 ± 12.01	42.64 ± 12.59	47.79 ± 11.71	<0.001
Gender					<0.001
Men (N/%)	20 (33.33)	53 ± 76.81	59 ± 85.51	107 ± 93.04	
Women (N/%)	40 (66.67)	16 ± 23.19	10 ± 14.49	8 ± 6.96	
WBC count (×10E9/L)	6.02 ± 1.27	5.71 ± 1.66	6.2 ± 2.79	7.53 ± 4.21	<0.001
HGB (g/L)	137.33 ± 11.70	135.19 ± 27.09	131.02 ± 21.03	115.05 ± 22.40	<0.001
PLT (×10E9/L)	269.92 ± 65.35	182.87 ± 86.25	170.06 ± 65.56	115.287 ± 75.58	<0.001
ALB (g/L)	N/A	42.59 ± 7.50	37.82 ± 4.85	34.11 ± 4.75	
AST (U/L)	19.68 ± 8.40	46.29 ± 51.07	616.17 ± 633.04	377.89 ± 549.94	<0.001
ALT (U/L)	8.95 ± 3.00	42.99 ± 40.66	1,016.46 ± 672.59	549.44 ± 807.16	<0.001
Tbil (μmol/L)	N/A	42.14 ± 72.63	118.72 ± 104.68	336.829 ± 138.73	<0.001
Cr (μmol/L)	58.39 ± 13.18	80.12 ± 27.65	70.96 ± 15.28	100 ± 285.72	<0.001
INR (No.)	N/A	1.33 ± 0.22	1.21 ± 0.20	2.38 ± 0.69	
PTA (%)	N/A	80.43 ± 12.95	78.86 ± 20.42	34.31 ± 8.60	<0.001
Fib (g/L)	N/A	3.24 ± 4.02	3.05 ± 4.17	1.53 ± 0.54	<0.001
AFP (ng/ml)	N/A	38.12 ± 97.76	121.93 ± 271.38	129.00 ± 223.62	
HBV-DNA (IU/ml)	N/A	1.63E+06 ± 1.07E+07	4.49E+07 ± 7.79E+07	7.69E+07 ± 5.88E+08	
HBsAg					
Positive (N/%)	N/A	19 (27.54%)	28 (40.58%)	25 (21.74%)	
Negative (N/%)	N/A	50 (72.46%)	41 (59.42%)	90 (78.26%)	
Liver cirrhosis					
Yes (N/%)	N/A	21 (30.43%)	9 (13.04%)	46 (40%)	
No (N/%)	N/A	48 (69.57%)	60 (86.96%)	69 (60%)	

HBV, hepatitis B virus; CHB, chronic hepatitis B; Cir, liver cirrhosis; HBV-flare, CHB patients with flare; ACLF, acute-on-chronic liver failure; WBC, white blood cell; HGB, hemoglobin; PLT, platelet; ALB, albumin; AST, aspartate transaminase; ALT, alanine transaminase; Tbil, total bilirubin; Cr, creatinine; INR, international normalized ratio; PTA, prothrombin time activity; Fib, fibrinogen; AFP, alpha fetoprotein; HBsAg, hepatitis B surface antigen; N/A, Not Applicable.

### Ethics Statement

This work was approved by the Ethics Committee of the Third Affiliated Hospital of Sun Yat-sen University [Guangzhou, China; ID: (2018) 02-384-01]. Each participant provided informed consent for participation. This work was conducted following the Declaration of Helsinki.

### Plasma Sample Collection

After gaining approval from the Ethics Committee of the Third Affiliated Hospital of Sun Yat-sen University, peripheral blood was sampled in the Third Affiliated Hospital of Sun Yat-sen University (Guangzhou, China) in line with standard protocols. Briefly, 10 ml of venous blood was drawn into ethylenediaamine tetra-acetic acid (EDTA)-coated vacuum tubes for a 30-min period under ambient temperature, followed by 10-min centrifugation at 3000 rpm and 4°C. The plasma sample supernatants were subsequently transferred into the RNase-free eppendorf (EP) tube and stored at -80°C.

### Exosome Isolation and Purification

For plasma samples, Total Exosome Isolation Kit (Cat # 4484450, Invitrogen, USA) was utilized to isolate plasma exosomes according to the manufacturer’s protocol. Briefly, this work processed plasma supernatants by 20-min centrifugation at 2,000 × g under ambient temperature in order to remove debris and cells. Then, the above supernatants were transferred into the new tube, followed by another 20-min centrifugation at 10,000 × g under ambient temperature to remove debris. The supernatants were transferred into new 1.5-ml tubes for exosome isolation. After resuspension of isolated exosomes resuspended in 0.22-μm filtered PBS could be used immediately or stored in a -80°C refrigerator.

### Nanoparticle Tracking Analysis

This work measured the size distribution of plasma exosomes in NTA by a laser particle size analyzer (Nanosight NS300, Malvern) according to the manufacturer’s software manual. Then, the nanoparticle numbers were explored by the origin software.

### Transmission Electron Microscopy

In transmission electron microscopy (TEM) analysis, this work placed plasma exosomes that were suspended in the particle-free PBS on the 200-mesh copper grid, followed by 2-min negative staining using 2% phosphotungstic acid. The plasma exosomes were dried in the air for a 15-min period, followed by TEM observation at 120 kV (FEI Tecnai G2 Spirit, Thermo Scientific, USA).

### Western Blot Analysis

To extract total protein from plasma exosomes, exosomes were first exposed to Proteinase K treatment and were collected by Radio Immunoprecipitation Assay (RIPA) lysis buffer that contained protease inhibitor cocktail for a 30-min period on ice, followed by 10-min centrifugation at 13,000 × g and 4°C. Thereafter, this work adopted Pierce^®^ BCA Protein Assay kit (Thermo Scientific, USA) in quantifying exosomal proteins. For the detection of the exosomal biomarker proteins, exosome lysates (30 μg) were separated on 12% tricine-Sodium dodecyl sulfate-polyacrylamide gel electrophoresis (SDS-PAGE), followed by electronic blotting on the 0.2-μM nitrocellulose (NC) membrane (Millipore). Later, Western blot (WB) assay was conducted with anti-CD63 (1:1,000, abcam, #ab134045), anti-CD9 (1:1,000, abcam, #ab263019), and anti-CD81 (1:1,000, abcam, #ab109201) antibodies. The Gel Imaging System (Syngene G: BOX F3, USA) was employed to observe signals.

### Exosome RNA Extraction and qRT-PCR

We collected exosomes from subjects in the discovery and validation cohorts. Later, RNA was extracted from plasma exosomes with TRIzol (Life Technologies, Cat# 15596018) in line with specific instructions, with synthesized cel-miR-39 RNA being introduced as spike-in standards to quantify the potential expression of sncRNAs more accurately in plasma exosome samples. miRNA First Strand cDNA was performed with a PrimeScript™ RT reagent Kit (Takara, Cat# RR037A) in line with specific instructions. By adopting TB Green^®^ Premix Ex Taq™ II (Takara, Cat# RR820A), qPCR procedure was carried out thrice. The sncRNA expression was normalized to cel-miR-39, and the expression of the genes was explored using the ΔΔCt method for the relative quantitation (RQ) of gene expression. **Supplementary Table S8** displays corresponding primers utilized in the present work.

### Small RNA Sequencing Library Preparation and High-Throughput Sequencing

For traditional small RNA-seq profiling, the plasma exosome RNA was purified from the discovery cohort (n = 13). By using the QIAseq^®^ miRNA Library Kit (QIAgen, Cat# 331505), small RNA-seq libraries were constructed with 2~10 ng of the prepared exosomal RNA in line with the manufacturer’s protocols. Before RNA-seq, 13 libraries with different indices were pooled at equal concentration. In addition, pooled libraries were sequenced on an Illumina 3000 analyzer by EPIBIOTEK Company (Guangzhou, China).

### Small Noncoding RNA Sequencing Data Analysis

The software SPORTS (version 1.1) (Shi et al., 2018) was applied to annotate the raw sncRNA-seq data with one mismatch tolerance. Briefly, the adapter sequences (AACTGTAGGCACCATCAAT) were removed from the raw data reads using cutadapt software (Martin, 2011). Afterward, clean reads were mapped against miRbase, mitochondrial tRNA database (mitotRNAdb), genomic tRNA database (GtRNAdb), rRNA and YRNA database of the National Center for Biotechnology Information (NCBI), piRNA database (including piRBase and piRNABank), and Ensembl ncRNA database using bowtie software. The reads derived from the same mature miRNA were summed by using the scratch-developed python script, so as to obtain the read count number of the mature miRNAs. Besides, only the sncRNAs with at least one read in at least 50% of the samples were retained, and the reads per million mapped reads (RPM) values for the sncRNA species were calculated.

### Identification of Differentially Expressed Genes

Using the DESeq2 package (Love et al., 2014), differentially expressed genes (DEGs) between two comparison groups, ACLF specimens (L) and normal specimens (N) and HBV-flare specimens (H) and normal specimens (N), were identified. The deemed DEGs were screened according to *P*-values <0.05 and |FC| ≥2. The heatmap was plotted using pheatmap software (version 1.0.10). In addition, the creation of other figures was performed using “ggplot2” R package in R software.

### tRNA-Derived Small RNA Correlation Analysis

We summed the fragment derived from the same tRNA to obtain the expression of tsRNA groups. The degree of correlation between these tsRNA categories was assessed using the Pearson correlation analysis, and subsequently, we plotted the expression distribution for the significantly associated tsRNA categories (*r* ^2^ > 0.56, *P* < 0.05).

### The Prediction of Parent tRNA Structure

The parent tRNA structure prediction was performed using RNAfold software (Gruber et al., 2008), and the results were visualized with foRNA (Kerpedjiev et al., 2015).

### Functional Enrichment Analysis

Gene Ontology (GO) and Kyoto Encyclopedia of Genes and Genomes (KEGG) enrichment analysis was performed *via* clusterProfiler package (Yu et al., 2012), and the significant top 10 terms were visualized. The miRNA and tsRNA target prediction software miRanda and TargetScan were used to predict the corresponding targets (Enright et al., 2003; Agarwal et al., 2015; Jehn et al., 2020), with the intersection of the two serving as the final predictions.

### Developing the Molecular Signatures

To develop the mi/ts/rsRNA (MTR-RNA) signature, only sncRNA species in the discovery cohort (n = 13) that were differentially expressed between the ACLF specimens (L) and normal specimens (N) and between HBV-flare specimens (H) and normal specimens were retained. To address the high dimensionality of the RNA-seq data, we applied LASSO LR to screen the candidate sncRNAs for the qPCR verification. The package “glmnet” was adopted for LASSO multinomial LR model (Friedman et al., 2010) to select the independent predictors. Considering the basal expression levels and differential expression folds of these 20 candidate sncRNAs in these 13 subjects that were based on the LR model, we finally obtained a set of 11 candidate sncRNAs. All validation cohort (n = 313) collected was randomized as training (70%) or validation (30%) set. Based on the qPCR data in the validation cohort, we constructed a multinomial LR model using the “nnet” package. The current work utilized the pROC package (Robin et al., 2011) to evaluate significant differences in the area under the receiver operating characteristic (ROC) curve (AUC) values.

### Statistical Analysis

For RNA-seq samples, biological repeats and statistical analyses were depicted in the *Materials and Methods* or Figure legends. Spearman’s rank correlation was conducted to obtain correlation coefficients (ρ). By adopting the log_2_-transformed scale, R package heatmap function was employed to generate heatmaps for the DEGs. Mann–Whitney U test and Student’s t-test were utilized to compare two groups, whereas Kruskal–Wallis test was applied to compare multiple groups. The statistical method of the clinical baseline table is as follows: the continuous variables were calculated to obtain median (minimum, maximum) and standard deviation with the frequency and percentage of individual variables being counted. For all analyses, the two-sided *P* < 0.05 stood for statistical significance, and statistical analysis was performed using R software (version 4.0.3).

## Results

### Genome-Wide Identification of Plasma-Derived Exosomal Small Noncoding RNAs in Hepatitis B Virus–Acute-on-Chronic Liver Failure

**Figure 1:**
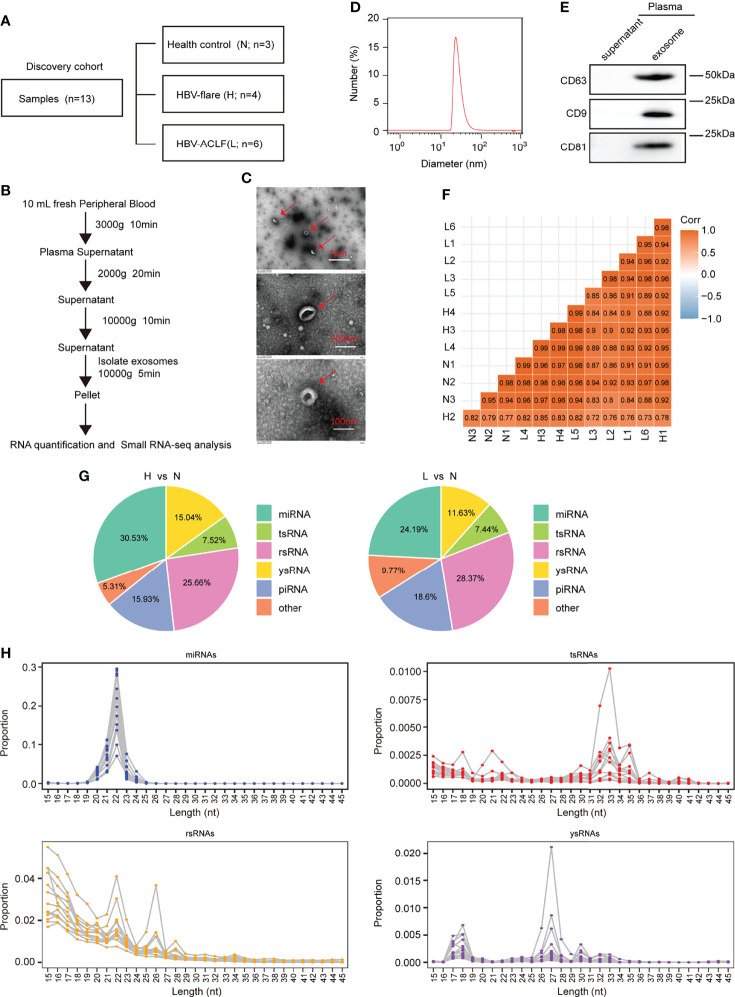
Profiling of sncRNAs in exosomes from patient plasma. **(A)** The samples included 3 healthy controls (marked as N), 4 HBV-flare patients (marked as H), and 6 HBV-ACLF patients (marked as L) for exosome small RNA-seq as the discovery cohort. **(B)** Exosome collection procedure from patient plasma. **(C)** TEM on plasma exosomes in ACLF patients (The scale bar is 1 μm, 200 nm, and 100 nm, respectively). **(D)** Nanoparticle tracking analysis (NTA) of exosomes isolated from ACLF patient plasma. **(E)** Western blot analysis for plasma exosomal protein. **(F)** Correlation of 13 sncRNA-seq. **(G)** sncRNA percentages between the two comparison groups: H vs. N and L vs. N. **(H)** The length distribution of the four types of sncRNAs, miRNAs, tsRNAs, rsRNAs, and ysRNAs. Each dot represents one plasma exosome sample. The Y-axis presented the read proportion within each sncRNA category.

To identify plasma-derived exosomal sncRNAs in HBV-ACLF, we performed small RNA-seq on plasma exosomes harvested in 13 discovery cohort participants, who included 3 normal subjects (marked as N), 4 CHB patients with flare (marked as H), and 6 HBV-ACLF patients (marked as L) (**Figures 1A, B**). This work conducted TEM, nanoparticle tracking analysis (NTA), and WB analyses on those isolated plasma exosomes using the exosomal protein marker CD63, CD9, and CD81, respectively (**Figures 1C–E**). The 3′ adapters were removed from the raw data reads, and then the low-quality sequences were further trimmed (**Supplementary Figure S1**). To evaluate the overall correlation of the 13 exosome RNA-seq data derived from different subjects, we performed Pearson correlation analysis on all ACLF, HBV-flare, and healthy control samples. The correlation for exosome RNAs from different human plasma-derived exosomes ranged from 0.72 to 0.99 (**Figure 1F**). Using the SPORTS (Shi et al., 2018) software, it could be found that the mapped genes were categorized into six groups, namely, miRNAs, tsRNAs, rsRNAs, ysRNA, piRNAs, and others (**Supplementary Figure S2**). We compared the expression of the annotated genes in the different samples to perform a genome-wide analysis of the small RNA expression profiles. The comparison group of H vs. N (|FC| ≥2; *P* < 0.05) contained 69 (30.53%) annotated miRNAs, 17 (7.52%) annotated tsRNAs, 58 (25.66%) annotated rsRNAs, 34 (15.04%) annotated ysRNAs, 36 (15.93%) annotated piRNAs, and 12 (5.31%) other types of sncRNAs (**Figure 1G**, left; **Supplementary Table S1**). The other comparison group was between L and N (|FC| ≥2; *P* < 0.05), which contained 52 (24.19%) annotated miRNAs, 16 (7.44%) annotated tsRNAs, 61 (28.37%) annotated rsRNAs, 25 (11.63%) annotated ysRNAs, 40 (18.6%) annotated piRNAs, and 21 (9.77%) other types of sncRNAs (**Figure 1G**, right; **Supplementary Table S1**). As expected, miRNAs were generally 20–24 nucleotides (nts) in length. However, the size patterns of tsRNAs, rsRNAs, and ysRNAs were more complicated with multiple distribution peaks (**Figure 1H**), suggesting the distinct biogenesis pathways and functional roles of noncanonical sncRNAs relative to miRNAs. Collectively, these results revealed that miRNAs and rsRNAs were predominant sncRNAs measured through sncRNA-seq; tsRNAs and ysRNAs also could be incorporated into exosomes and secreted into the extracellular environment.

### RNA Sequencing Analysis Reveals a Batch of Dysregulated miRNAs in Hepatitis B Virus–Acute-on-Chronic Liver Failure

**Figure 2:**
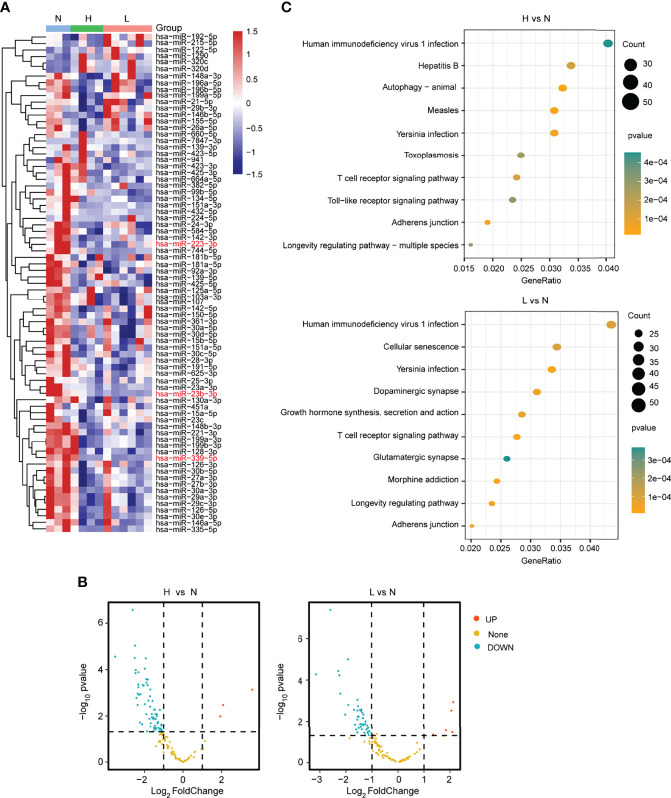
Differential expression analysis of miRNAs from plasma. **(A)** The heatmap showed the differentially expressed miRNAs in the L, H, and N group samples. **(B)** The volcano plot presented the differentially or non-differentially expressed miRNAs between the H vs. N and L vs. N comparison groups. **(C)** The KEGG pathway analysis for the differentially expressed miRNAs in the above two comparison groups.

To identity the differentially expressed miRNAs responsive to the HBV-ACLF, we further explored the small RNA-seq data. The heatmaps and volcano diagram revealed that there were 3 miRNAs upregulated and 65 miRNAs downregulated (|FC| ≥2; *P* < 0.05) in the group of H vs. N. In the other group (L vs. N), 5 miRNAs were upregulated and 46 miRNAs were downregulated (|FC| ≥2; *P* < 0.05) (**Figures 2A, B**; **Supplementary Table S2**). Furthermore, the KEGG analysis revealed that the T-cell receptor signaling pathway was well enriched in both comparison groups. Especially, the hepatitis B pathway and the cellular senescence pathway were dramatically enriched in the comparison groups H vs. N and L vs. N, respectively (**Figure 2C**; **Supplementary Table S3**). Based on all of the above findings, analyzing RNA-seq data provides a comprehensive and *bona fide* miRNA regulatory network.

### The Molecular Signature Composed of Noncanonical Small Noncoding RNAs

**Figure 3:**
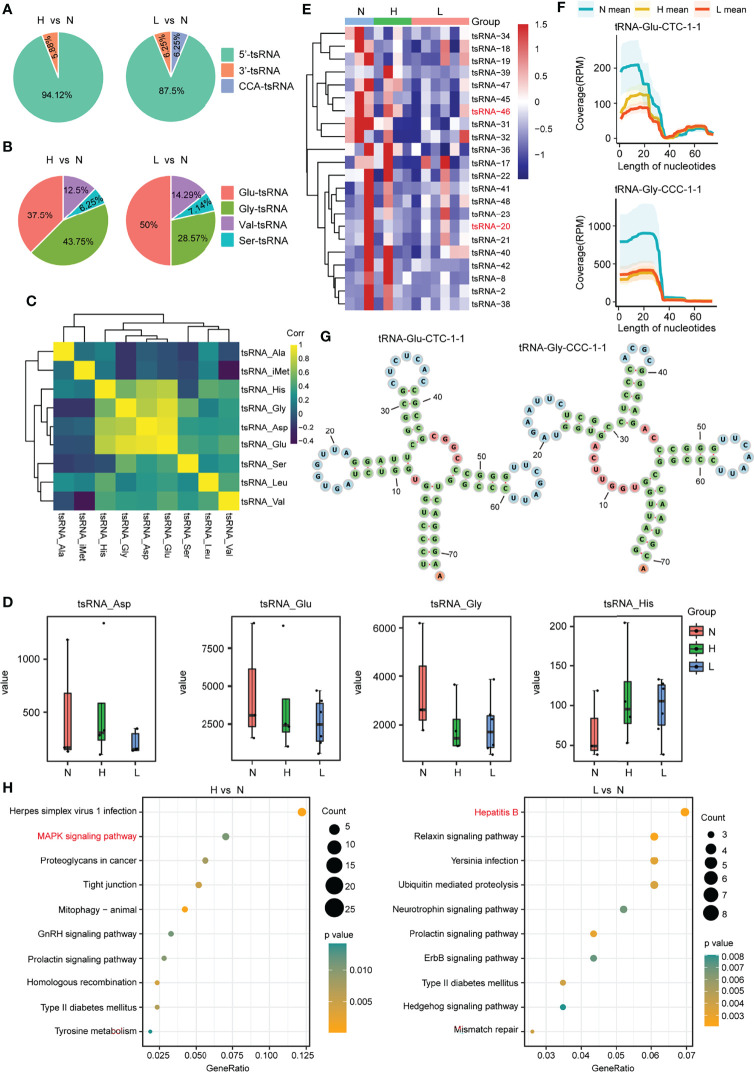
tsRNA differential expression analysis within plasma exosomes. **(A)** Percentage of each type of tsRNAs in the above two comparison groups. **(B)** Percentage of 5′-tsRNAs generated from Glu-, Gly-, Val-, and Ser-tRNA. **(C)** tsRNA coexpression patterns among plasma exosomes from the discovery set. Spearman’s rank correlation coefficient. tsRNA-Gly, tsRNA-His, tsRNA-Glu, and tsRNA-Asp showed strong and positive relation mutually. **(D)** Expression profiles for tsRNA-Glu, tsRNA-Asp, tsRNA-His, and tsRNA-Gly among the N, H, and L subjects. **(E)** Heatmap showing differential tsRNA expression among L, N, and H sets. **(F)** The coverage profile of the plasma exosomal tsRNA-20 and tsRNA-46 sequences along tRNA-Gly-CCC-1-1 and tRNA-Glu-CTC-1-1, respectively. Solid curves presenting average RPM of N, H, and L groups. RPM, reads per million; nt, nucleotide. Colored band stood for 95% CI. tsRNA-20 and tsRNA-46 sequences were obtained in tRNA 5′-terminal. **(G)** The tRNA secondary structure revealed the representative individual tsRNA. **(H)** The KEGG pathway analysis for the differentially expressed tsRNAs in the above two comparison groups.

Then, we focus on the noncanonical sncRNA studies. Between the two comparison groups, we found that the mature tRNA 5-terminal-derived 5′-tsRNA was the most abundant tsRNA in exosomes, accounting for 94.12% and 87.5%, respectively, 3′-tsRNA was 5.88% in H vs. N group, whereas both 3′-tsRNA and CCA-tsRNA were 6.25% in the comparison group L vs. N (**Figure 3A**; **Supplementary Figure S4**). Moreover, by exploring the origin of these most abundant tsRNA, we found that the 5′-tsRNA was derived from four types of tRNAs (Glu-, Gly-, Val-, and Ser-tRNA) (**Figure 3B**). In addition, this work also examined tsRNA coexpression profiles within plasma exosomes in the discovery cohort by grouping all tsRNAs in line with their corresponding parental tRNA types. We discovered that the expression of the tsRNA derived from the tRNAs of aspartic acid (tsRNA-Asp), glutamic acid (tsRNA-Glu), glycine (tsRNA-Gly), and histidine (tsRNA-His) showed potent positive relation mutually (*r* ^2^ ranged from 0.56 to 0.94) (**Figure 3C**), suggesting that these tsRNAs may share biological pathways. Interestingly, among the above four tsRNA groups, compared with the health control group, tsRNA-Glu and tsRNA-Gly were the tsRNA groups that were downregulated in the ACLF and HBV-flare patients, while tsRNA-Asp and tsRNA-His were the tsRNA groups that were upregulated in the HBV-flare patients in comparison with the healthy controls (*P* < 0.05) (**Figure 3D**). The heatmap showed that the 17 tsRNAs were all downregulated (|FC| ≥2; *P* < 0.05) in the comparison group of H vs. N. In the other group (L vs. N), 16 tsRNAs were downregulated (|FC| ≥2; *P* < 0.05) (**Figure 3E**; **Supplementary Table S4**).

We further mapped the individual tsRNA-20 (derived from tRNA-Gly-CCC-1-1) and tsRNA-46 (derived from tRNA-Glu-CTC-1-1) species to their corresponding parental tRNAs and obtained an appraisal that these tsRNAs were not in a random fragmentation pattern (**Figures 3F, G**), indicating that the biogenesis of these sncRNAs was highly regulated. In addition, the KEGG analysis showed that the Mitogen‑activated protein kinase (MAPK) signaling pathway and the hepatitis B pathway were significantly enriched in the comparison group of H vs. N or L vs. N, respectively (**Figure 3H**; **Supplementary Table S5**). Consequently, we obtained an overview of tsRNAs, the noncanonical sncRNAs, in response to HBV-ACLF.

### Dysregulated rsRNAs in Hepatitis B Virus–Acute-on-Chronic Liver Failure

**Figure 4:**
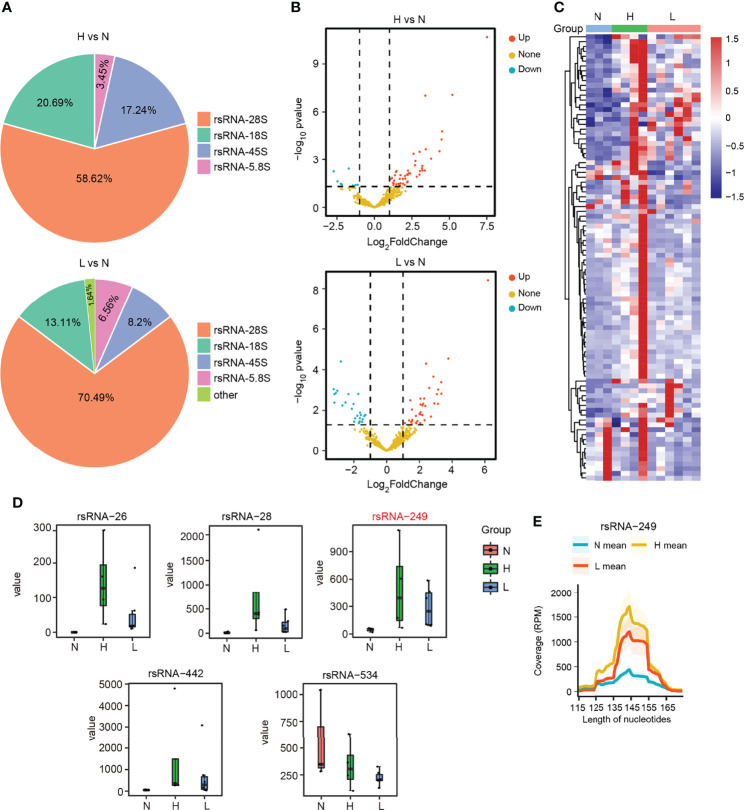
The dysregulated rsRNAs in HBV-ACLF. **(A)** Percentage of each type of rsRNAs between the comparison groups H vs. N and L vs. N. **(B)** The volcano plot showed the differentially or non-differentially expressed rsRNAs in the above two comparison groups. **(C)** The heatmap revealed the differentially expressed rsRNAs in the N, H, and L subjects. **(D)** The expression profile of rsRNA-26, rsRNA-28, rsRNA-249, rsRNA-442, and rsRNA-534 among the N, H, and L subjects. **(E)** The coverage profile of the rsRNA-249 sequences of plasma exosomal along rRNA-28s.

To globally explore the dysregulated rsRNAs, also as noncanonical sncRNAs, in the HBV-ACLF patients, we performed small RNA transcriptomes with the discovery cohort. The SPORTS (Shi et al., 2018) analysis revealed that the rsRNAs consisted of the following five types: rsRNA derived from rRNA-18S (rsRNA-18S), rsRNA derived from rRNA-45S (rsRNA-45S), rsRNA derived from rRNA-28S (rsRNA-28S), rsRNA derived from rRNA-5.8S (rsRNA-5.8S), and rsRNA derived from other types of rRNA. Among these five types of rsRNAs, rsRNA-28S was the most abundant in the two compared groups (**Figure 4A**; **Supplementary Figures S4**–**S6**). According to the differential expression analysis, 49 rsRNAs were upregulated and 9 rsRNAs were downregulated (|FC| ≥2; *P* < 0.05) in the group of H vs. N. In the other group (L vs. N), 36 rsRNAs were upregulated and 25 rsRNAs were downregulated (|FC| ≥2; *P* < 0.05) (**Figures 4B, C**; **Supplementary Table S6**). Among the above rsRNAs, there were top 6 differentially expressed rsRNAs in the 13 subjects (|FC| >3; RPM > 1,000; *P* < 0.05). It could be found that rsRNA-26, rsRNA-28, rsRNA-249, and rsRNA-442 markedly increased among HBV-flare and ACLF cases relative to normal controls (*P* < 0.05), whereas rsRNA-534 showed more downregulated expression in the ACLF and HBV-flare cases compared with normal controls (*P* < 0.05) (**Figure 4D**). In addition, we further mapped the individual rsRNA-249 (derived from rRNA-28S) species to its corresponding parental rRNA and identified that the rsRNA was not a random fragmentation model (**Figure 4E**), indicating that the biogenesis of sncRNAs is also regulated with high precision.

### The Performance of the MTR-RNA Signature in the Validation Cohort

**Figure 5:**
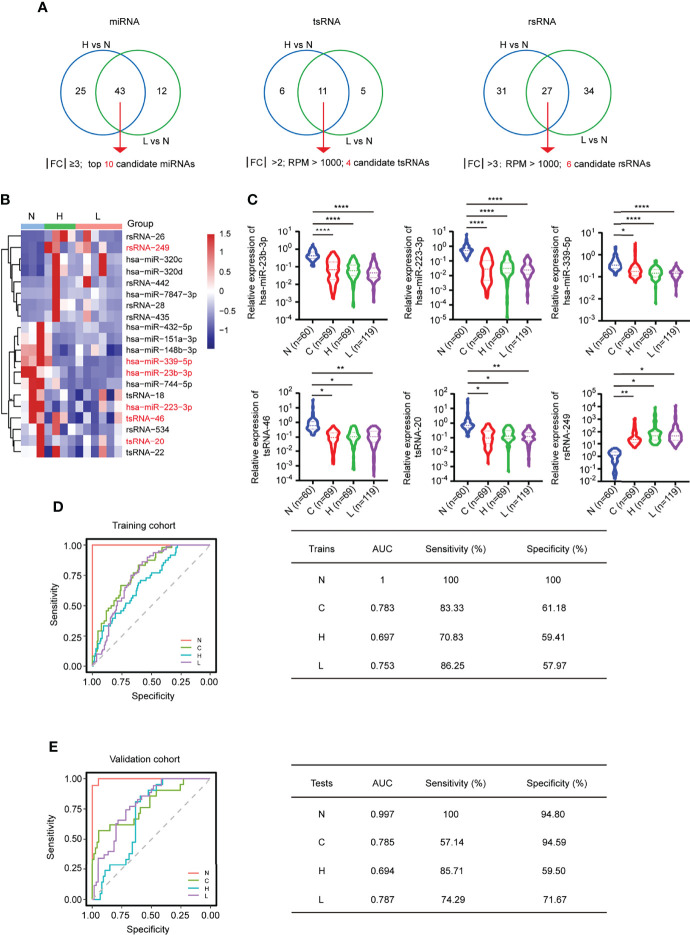
MTR-RNA signature in predicting the validation set. **(A)** The Venn diagram showed the significantly differentially expressed sncRNAs between the comparison groups H vs. N and L vs. N. **(B)** Heatmap presenting sncRNA species in the MTR-RNA signature for the discovery set. **(C)** RT-qPCR analyses of exosomal hsa-miR-23b-3p, hsa-miR-223b-3p, hsa-miR-339-5p, tsRNA-20, tsRNA-46, and rsRNA-249 in the four groups (N = 60, C = 69, H = 69, and L = 115). Data are represented as mean ± SEM. *P < 0.05, **P < 0.01 and ****P < 0.001. **(D, E)** ROC curve for MTR-RNA index used to distinguish ACLF cases from healthy controls and HBV-flare cases from healthy controls of the training set **(D)** and the validation set, respectively **(E)**. The left represented the ROC curve. The right represented the model assessment. AUC, area under the ROC curve.

By further intersecting the differentially expressed sncRNAs in the two comparison groups (H vs. N; L vs. N), we identified 20 overlapping sncRNAs as candidate genes, named the MTR-RNA, including 10 miRNAs (|FC| ≥3; *P* < 0.05), 4 tsRNAs (|FC| >2; RPM > 1,000; *P* < 0.05), and 6 rsRNAs (|FC| >3; RPM > 1,000; *P* < 0.05) (**Figures 5A** **, B**; **Supplementary Table S7**). Next, we identified the potential candidate MTR-RNAs according to the RPM reads of characteristic molecules in sncRNA-seq based on the LASSO LR model analysis, and among the 20 candidate sncRNAs, 11 sncRNAs were selected for further investigation (**Supplementary Figures S7A–D**). In total, the validation cohort (n = 313) including healthy controls (n = 60), HBV-associated cirrhosis or CHB patients (n = 69), CHB patients with flare (n = 69), and HBV-ACLF patients (n = 115) was recruited to evaluate the levels of the 11 MTR-RNAs by RT-qPCR (**Figure 5C**; **Table 1**). Six (hsa-miR-23b-3p, hsa-miR-223-3p, hsa-miR-339-5p, tsRNA-20, tsRNA-46, and rsRNA-249) of the 11 sncRNAs met the predetermined threshold of significant expression level (**Figure 5C**). In the present study, the validation cohort (n = 313) was randomized as training (70%) or validation (30%) phase, of them, the training phase was carried out for assessing the as-identified MTR-RNA signature, whereas the validation phase was utilized for testing the chosen MTR-RNAs by using another cohort. According to ROC analysis, our as-constructed MTR-RNA signature performed well among HBV-ACLF cases. By adopting the best threshold of the MTR-RNA determined in the training cohort, the sensitivity and specificity of MTR-RNA were 86.25% and 57.97%, respectively, and the AUC was 0.753 for the HBV-ACLF patients (**Figure 5D**). Using the best MTR-RNA index threshold acquired based on ROC analysis, HBV-ACLF cases from the validation dataset were distinguished from normal subjects with MTR-RNA signature (AUC = 0.787), and the specificity and sensitivity were 71.67% and 74.29%, respectively (**Figure 5E**). Amusingly, HBV-flare cases, patients with acute exacerbations of CHB, from the validation dataset were distinguished from normal subjects with MTR-RNA signature (AUC = 0.694), and the specificity and sensitivity were 85.71% and 59.50%, respectively. The MTR-RNA signature (AUC = 0.785) for patients with CHB/cirrhosis had 57.14% sensitivity and 94.59% specificity. Specially, the normal subjects from the validation dataset were distinguished from patients with MTR-RNA signature (AUC = 0.997) with extremely high specificity (100%) and sensitivity (94.80%) (**Figure 5E**). Compared with Total bilirubin (TB) and prothrombin time activity (PTA), which are characteristic of current traditional clinical guidelines, the MTA-RNA signature provides an auxiliary diagnostic function to a certain extent (**Supplementary Figure S8**).

Subsequently, we turned to bioinformatics to explore the function of the dysregulated MTR-RNA. The KEGG pathway analysis for the miRNAs (hsa-miR-23b-3p, hsa-miR-223-3p, and hsa-miR-339-5p) and tsRNAs (tsRNA-20 and tsRNA-46) showed that the MTR-RNA played a potential role in metabolism-, proliferation-, and apoptosis-related pathways (**Supplementary Figures S9A, B**; **Supplementary Tables S3**, **S5**). Therefore, the MTR-RNA signature could distinguish HBV-ACLF cases from normal controls partially, thereby making it possible for transformation.

## Discussion

**Figure 6:**
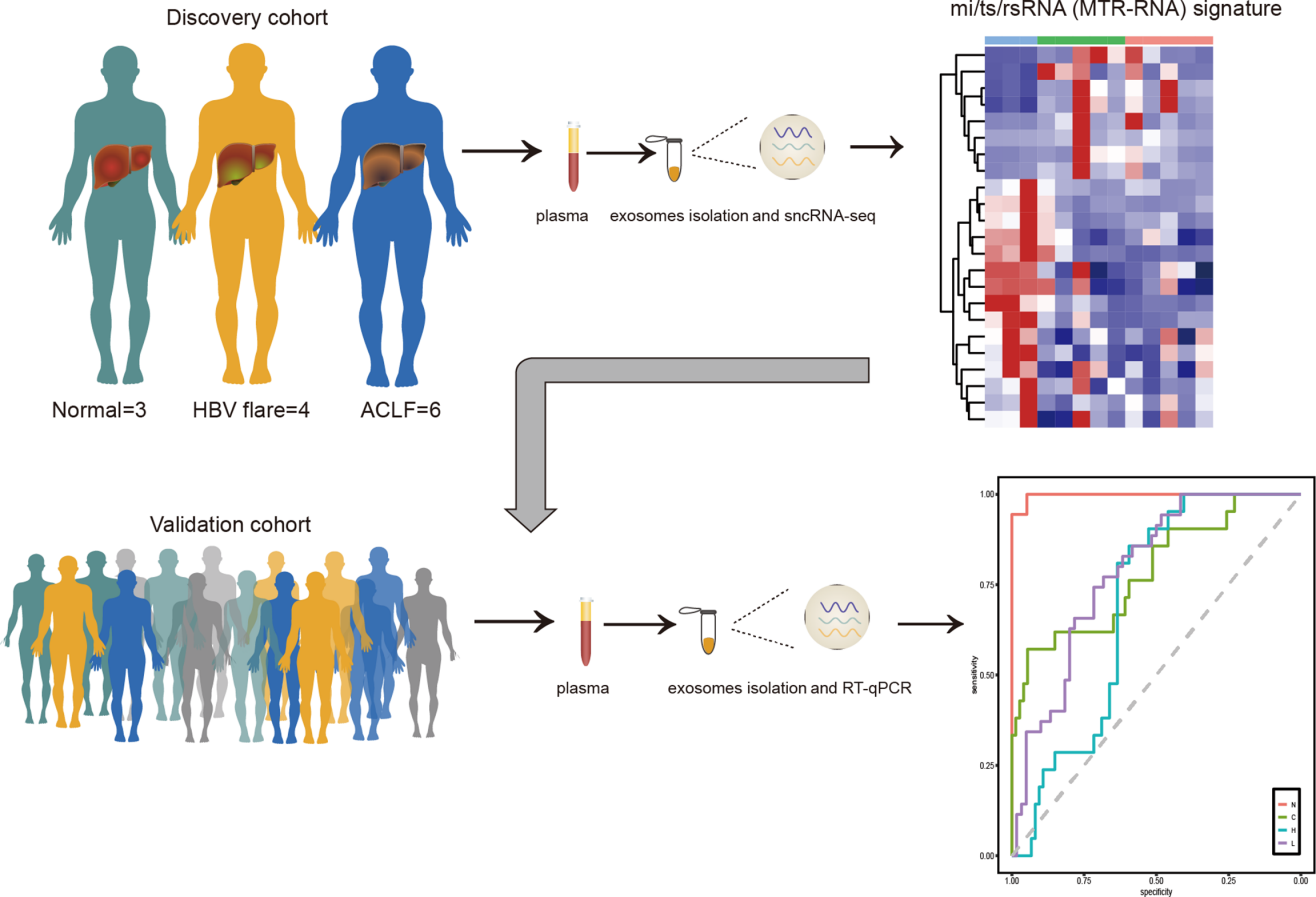
The MTR-RNA signature in HBV-ACLF. Plasma exosome mi/ts/rsRNA (MTR-RNA) expression of the discovery cohort subjects was profiled by sncRNA-seq. The molecular signature containing mi/ts/rsRNAs was developed to discriminate between healthy controls (N), HBV-associated cirrhosis or CHB patients (C), CHB patients with flare (H), and HBV-ACLF subjects (L).

This study characterized six sncRNAs (here named MTR-RNAs) by sncRNA-seq of plasma exosomes collected from HBV-ACLF cases. We established a noninvasive MTR-RNA biomarker signature that incorporated hsa-miR-23b-3p, hsa-miR-223-3p, hsa-miR-339-5p, tsRNA-20, tsRNA-46, and rsRNA-249, and it might be used to diagnose HBV-ACLF and to predict the benefits gained from early treatment (**Figure 6**).

Liquid biopsy has been developed as a strategy to diagnose diseases including cancer, and it has attracted wide attention recently. This work adopted high-throughput sequencing (HTS) for the differential sncRNA expression analysis. tsRNAs in exosomes are investigated in some published studies (Zhu et al., 2019; Wang et al., 2020a; Li et al., 2022). As is well known, RNA modifications in tsRNAs can interfere with adapter ligation and reverse transcription processes during small RNA library construction and thus prevent the detection of tsRNAs bearing these modifications. Researchers have made efforts to develop special experimental methods to overcome this limitation (Cozen et al., 2015). Recently, Shi et al. (2021) have developed a novel method, PANDORA-seq, to efficiently remove the modifications on tRNA, rRNA, and yRNA and have engineered both the 5′ and 3′ ends of the library fragments so that the linker conditions (i.e., 5’phosphate, 3’hydroxyl) can be met (Shi et al., 2022). In the future, using this new sequencing technology, it will unearth more modified noncanonical sncRNAs for us and will provide us with more reliable and real-world small RNAs. This is the first report of plasma exosome-derived miRNA and noncanonical sncRNAs (tsRNA and rsRNA) as the biomarkers for HBV-ACLF. Compared with TB and PTA, one of the classic indicators in traditional clinical guidelines, we found that the combination of our current MTR-RNA signature molecule can increase the reliability of diagnosis to a certain extent. Recently, Luo et al. (2022) had reported the establishment of a Chinese standard for the diagnosis of HBV-ACLF (COSSH-ACLF) and a prognostic stratification scoring system suitable for the hepatitis B population by conducting a prospective, multicenter, open-label large cohort study. Using the large cohort of the COSSH Open Study, they found that TB, INR, ALT, and serum ferritin (SF) were the best predictors of ACLF within 7 days. More prospective randomized trials should be conducted to validate whether our as-constructed sncRNA signature performs well in the clinic. Likewise, for the future, we need to compare the sensitivity and specificity of these new MTR-RNA molecules using the COSSH-ACLF scoring criteria. Particularly, the performance of sncRNA signature in distinguishing preclinical ACLF was assessed in a nested case-control study by prospectively collecting plasma exosomes from patients with ACLF and at-risk controls.

Similar to miRNAs, tsRNAs also bind to mRNA or the translation initiation complex to modulate gene levels. In this study, the tsRNA-20 and tsRNA-46 are produced by the 5′ ends of tRNAs and downregulated in HBV-ACLF and HBV-flare. This work explored the targets of tsRNA-20 and tsRNA-46 according to bioinformatics analysis. The KEGG pathway analysis for these two tsRNAs illustrated that the amino acid metabolism and the peroxisome proliferator-activated receptor (PPAR) signaling pathway were enriched. It is well known that ACLF is strongly related to metabolic disorder and damage (Zaccherini et al., 2021), and that PPARs are tightly associated with cell growth, apoptosis, differentiation, inflammatory response, and energy metabolism (Wang et al., 2020b). These suggest that these two tsRNAs possibly have essential effects on ACLF, while more investigations are needed to explore the underlying mechanism.

Thus far, in humans and mice, the known functional rsRNAs derived from rRNAs mainly participate in normal physiological processes (Natt et al., 2019) and mouse embryonic stem cells (mESC) differentiation (Shi et al., 2021). This present study identified and uncovered a batch of abnormally regulated rsRNAs. In particular, this study was the first to find that rsRNA-249 derived from rRNA-28S was significantly upregulated in human HBV-ACLF. Our findings suggest shared HBV-ACLF mechanisms between the HBV infection and the biogenesis of rsRNA, providing novel insights about the interplay in ACLF disease.

In conclusion, we developed a novel noninvasive biomarker HBV-ACLF-based sncRNA signature in plasma exosomes to diagnose CHB in patients with flare and HBV-ACLF. This diagnostic system was convenient and reliable. Particularly, it may be used to predict preclinical ACLF cases potentially benefiting from early adjuvant therapy.

## Data Availability Statement

The datasets presented in this study can be found in online repositories. The small RNA sequencing data that support the findings of this study have been deposited in NCBI Gene Expression Omnibus (GEO; https://www.ncbi.nlm.nih.gov/geo/) under accession number GSE202557. All the data analysis results have been provided in the supplementary files.

## Ethics Statement

The studies involving human participants were reviewed and approved by Third Affiliated Hospital of Sun Yat-sen University Ethics Committee (Guangzhou, China; ID: [2018] 02-384-01). The patients/participants provided their written informed consent to participate in this study.

## Author Contributions

YC, WX and MY conceived and designed the entire project. YC designed and supervised the research. WX, MY, YW, YJ, XL, XZ, FY, and YC performed the experiments and/or data analyses. WX, MY, YW, YJ, XL, XZ, FY, provided the study materials or patients. WX and MY performed the transcriptome-wide data analyses. WX and YC contributed reagents and/or grant support. WX, MY, YW, YJ, XL, XZ, FY, and YC wrote and revised the paper. All authors discussed the results and commented on the manuscript. All authors contributed to the article and approved the submitted version.

## Funding

This work was supported in part by the National Natural Science Foundation of China (32000892 and 81773176); the Science and Technology Planning Project of Guangdong Province, China (2019B020228001). This research was supported in part by the Guangdong Basic and Applied Basic Research Foundation (2019A1515110166) China Postdoctoral Science Foundation (232600) and Science and Technology Program of Guangzhou, China (201804010474).

## Conflict of Interest

The authors declare that the research was conducted in the absence of any commercial or financial relationships that could be construed as a potential conflict of interest.

## Publisher’s Note

All claims expressed in this article are solely those of the authors and do not necessarily represent those of their affiliated organizations, or those of the publisher, the editors and the reviewers. Any product that may be evaluated in this article, or claim that may be made by its manufacturer, is not guaranteed or endorsed by the publisher.

## Abbreviations

sncRNA, small noncoding RNA; miRNA, mircoRNA; tsRNA, tRNA-derived small RNA; rsRNA, rRNA-derived small RNA; ysRNA, yRNA-derived small RNA; HBV, hepatitis B virus; ACLF, acute-on-chronic liver failure; MTR-RNA, mi/ts/rsRNA; CHB, chronic hepatitis B; SAE, severe acute exacerbation; EV, extracellular vesicle; HCC, hepatocellular carcinoma; tRF, tRNA-derived fragment; tiRNA, tRNA halve; sncRNA-seq, small noncoding RNA sequencing; HBV-flare, CHB patients with flare; Cir, liver cirrhosis; TEM, transmission electron microscopy; NTA, nanoparticle tracking analysis; WB, Western blot; SDS-PAGE, SDS-polyacrylamide gel electrophoresis; NC, nitrocellulose; RQ, relative quantitation; mitotRNAdb, mitochondrial tRNA database; GtRNAdb, genomic tRNA database; NCBI, National Center for Biotechnology Information; RPM, reads per million mapped reads; DEG, differentially expressed gene; nt, nucleotide; HTS, high-throughput sequencing; ROC, receiver operating characteristic; AUC, area under the ROC curve; INR, international normalized ratio; ULN, upper limit of normal; Tbil, total bilirubin; PTA, prothrombin time activity; HBsAg, hepatitis B surface antigen; ALB, albumin; Fib, fibrinogen; WBC, white blood cell; Cr, creatinine; HGB, hemoglobin; PLT, platelet; AFP, alpha fetoprotein; ALT, alanine transaminase; AST, aspartate transaminase.
