# Supplementary material for: Plasma-Derived Exosomal SncRNA as a Promising Diagnostic Biomarker for Early Detection of HBV-Related Acute-on-Chronic Liver Failure
Source: Front Cell Infect Microbiol. 2022 Jul 7;12:923300. doi: 10.3389/fcimb.2022.923300 (PMC9301338; doi:10.3389/fcimb.2022.923300)
Supplement: Supplementary file 1 [file DataSheet_1.docx]

**Plasma-derived exosomal sncRNAs as a promising diagnostic biomarker for early dectection of HBV-related acute-on-chronic liver failure**

Wenli Xu^†^, Mingxue Yu^†^, Yuankai Wu, Yusheng Jie, Xiangyong Li, Xinxin Zeng, Fangji Yang and Yutian Chong^*^

^*^**Corresponding author.** **Email:**

chongyt@mail.sysu.edu.cn

***Supplementary Material***

**Figure S1: Mapping of overall length displaying relative sncRNA reads distributions in 13 exosome samples in small RNA-seq…………………………….3**

**Figure S2: Results presenting the dynamic sncRNAs landscape measured through 13 exosome samples in small RNA-seq………………………………………………4**

**Figure S3: Results showing the dynamic tsRNAs landscape measured in discovery cohort………………………………………………………………………………….5**

**Figure S4: Mapping of overall length displaying relative rsRNA read distributions in 13 exosome samples in small RNA-seq……………………………………………6**

**Figure S5: Results revealing the dynamic rsRNAs landscape measured in discovery cohort………………………………………………………………………7**

**Figure S6: Results showing the dynamic rsRNAs landscape derived from rRNA-28s and rRNA-18s detected in discovery cohort……………………………………8**

**Figure S7: The construction of a molecule signature model for candidate sncRNAs in the RNA-seq data from the discovery cohort…………………………………….9**

**Figure S8: Comparison of MTA-RNA signature molecules with current traditional clinical guidelines for TB and PTA, respectively…………………………………10**

**
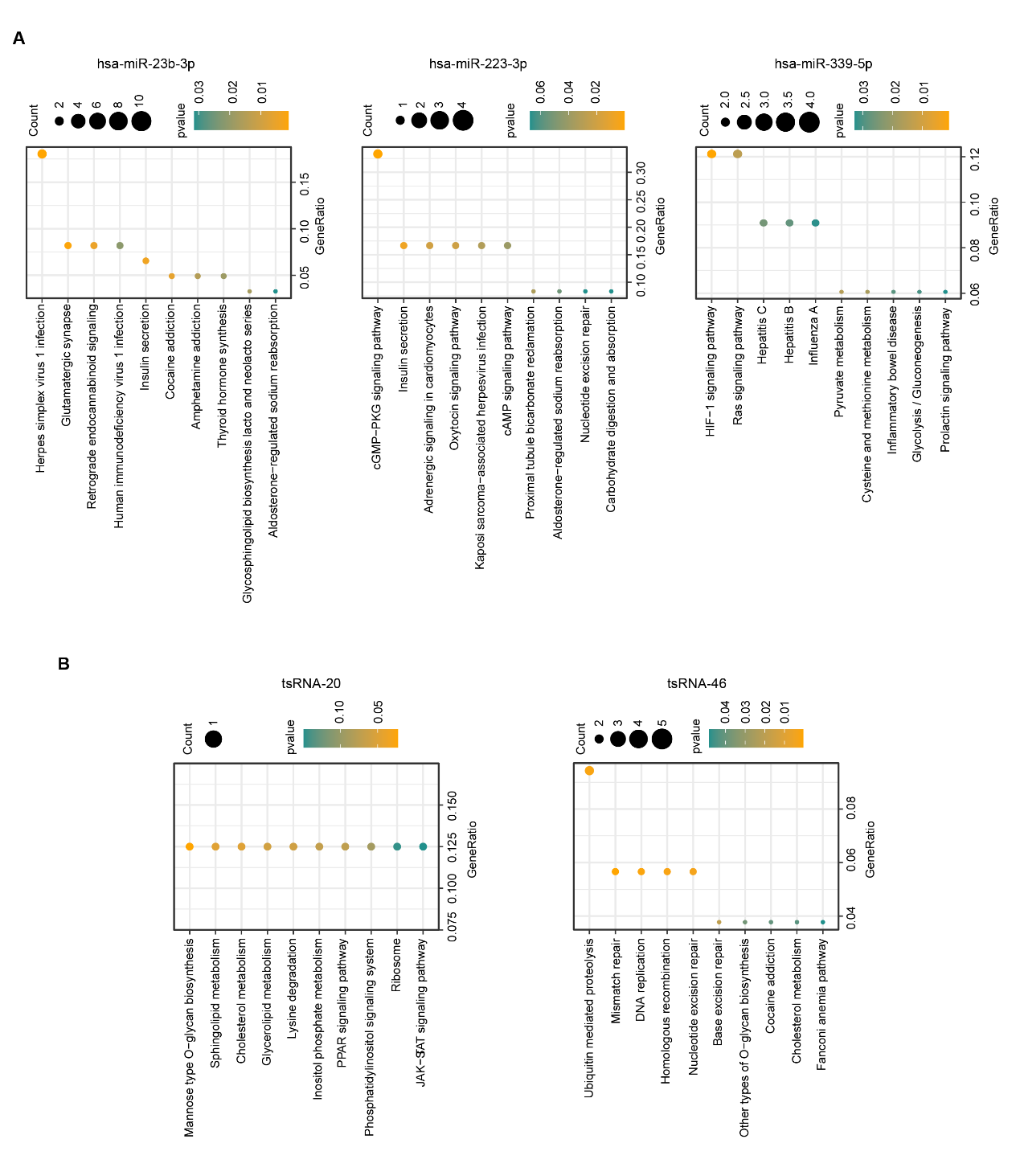
**

**Figure S9: The KEGG pathway analysis for the MTR-RNA signature including hsa-miR-23b-3p, hsa-miR-223-3p and hsa-miR-339-5p (A), and tsRNA-20 and tsRNA-46 (B)………………………………………………………………………...11**

**Table S1: Transcripts differentially expressed identified by the sncRNA-seq analysis.**

**Table S2: The differentially expressed miRNAs were analyzed by sncRNA-seq.**

**Table S3: Using miRanda and TargetScan softwares to predicte miRNA potential targets.**

**Table S4:** **The differentially expressed tsRNAs were analyzed by sncRNA-seq.**

**Table S5: Using miRanda and TargetScan softwares to predicte tsRNA potential targets.**

**Table S6: The differentially expressed rsRNAs were analyzed by sncRNA-seq.**

**Table S7: List of 20 sncRNA candidates used in this study.**

**Table S8: Sequences of primers used in this study.**

**Supplementary Figures**

**
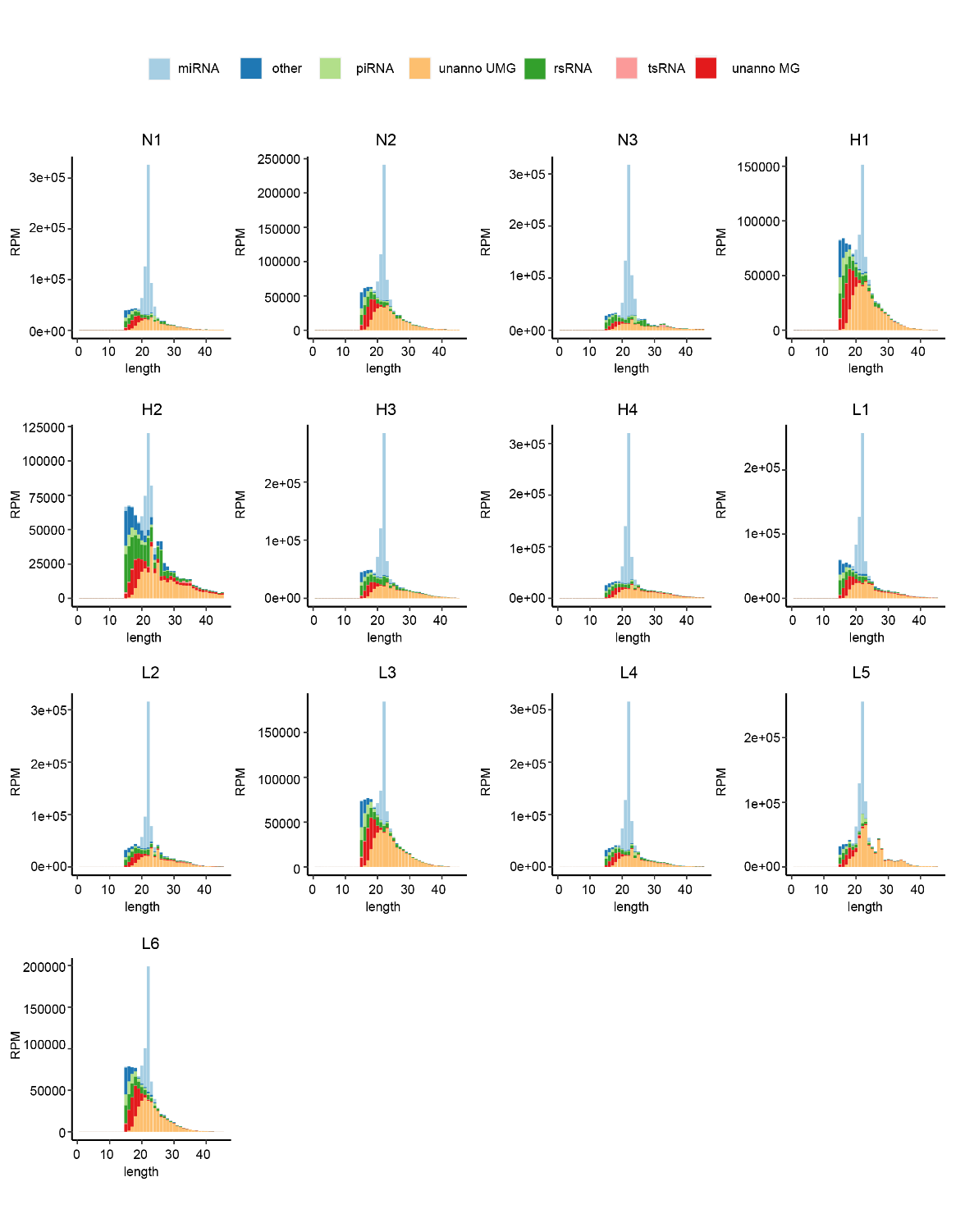
**

**Figure S1:** Mapping of overall length displaying relative sncRNA reads distributions in 13 exosome samples in small RNA-seq.


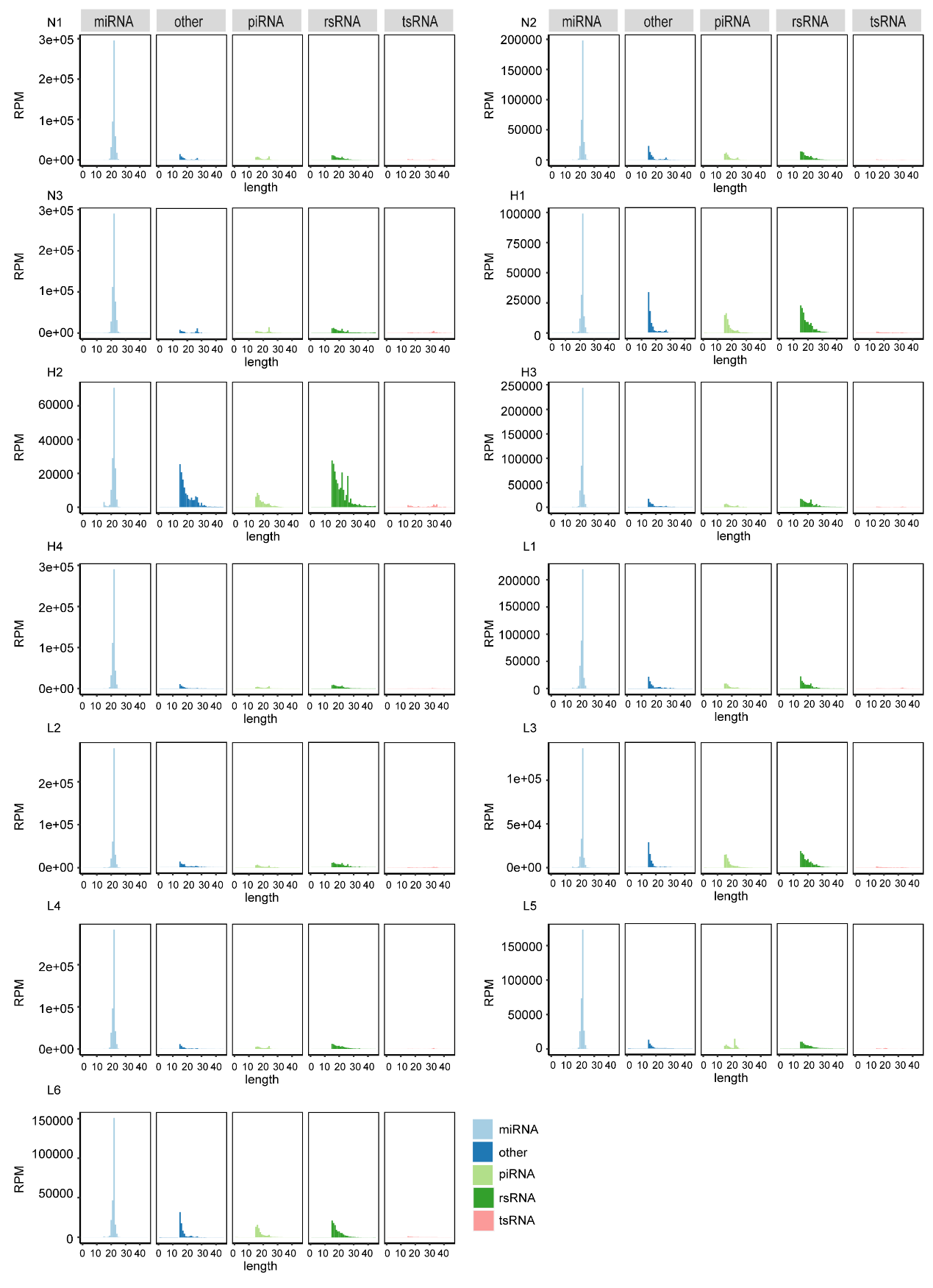


**Figure S2:** Results presenting the dynamic sncRNAs landscape measured through 13 exosome samples in small RNA-seq.

**
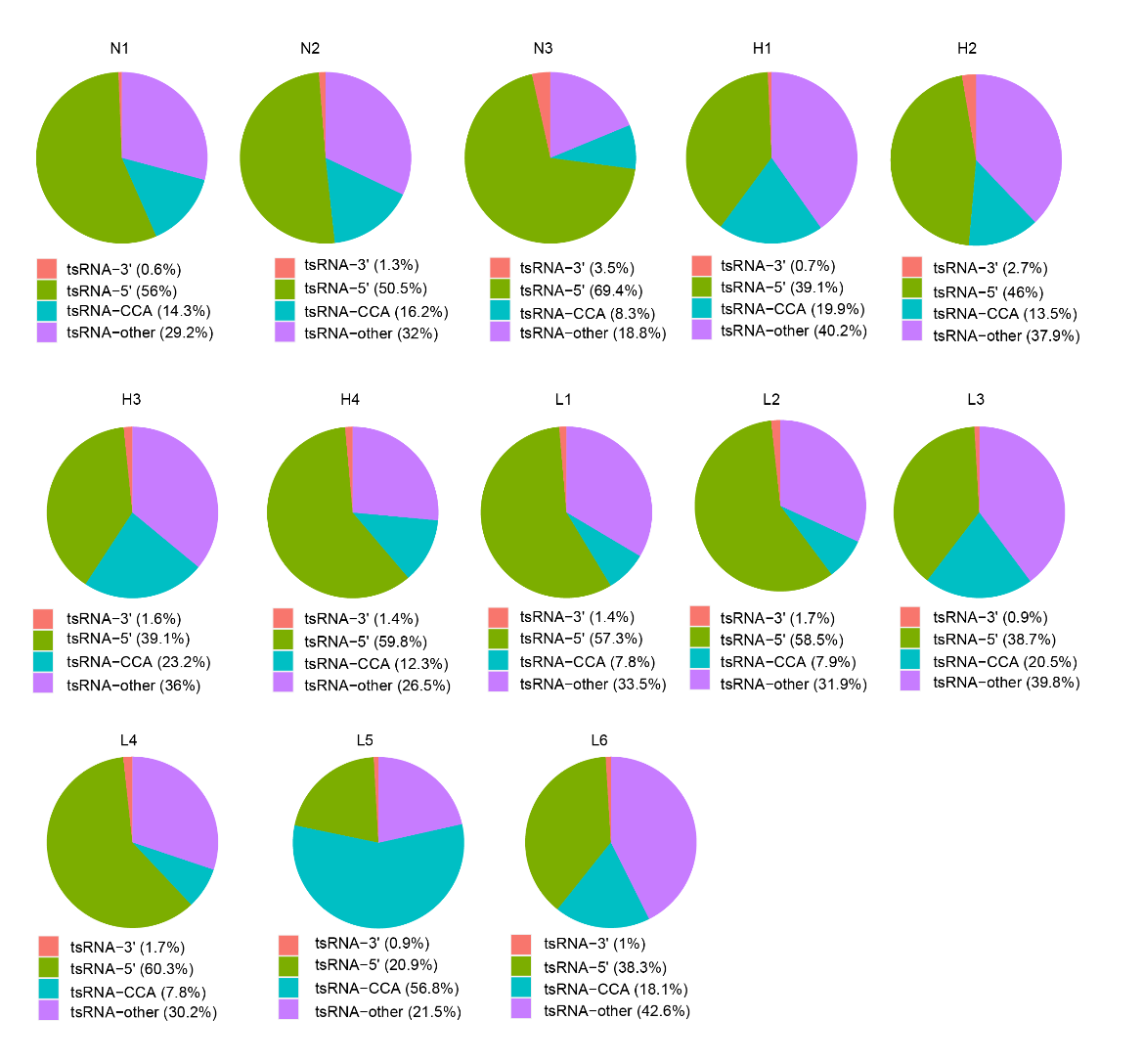
**

**Figure S3:** Results showing the dynamic tsRNAs landscape measured in discovery cohort.

**
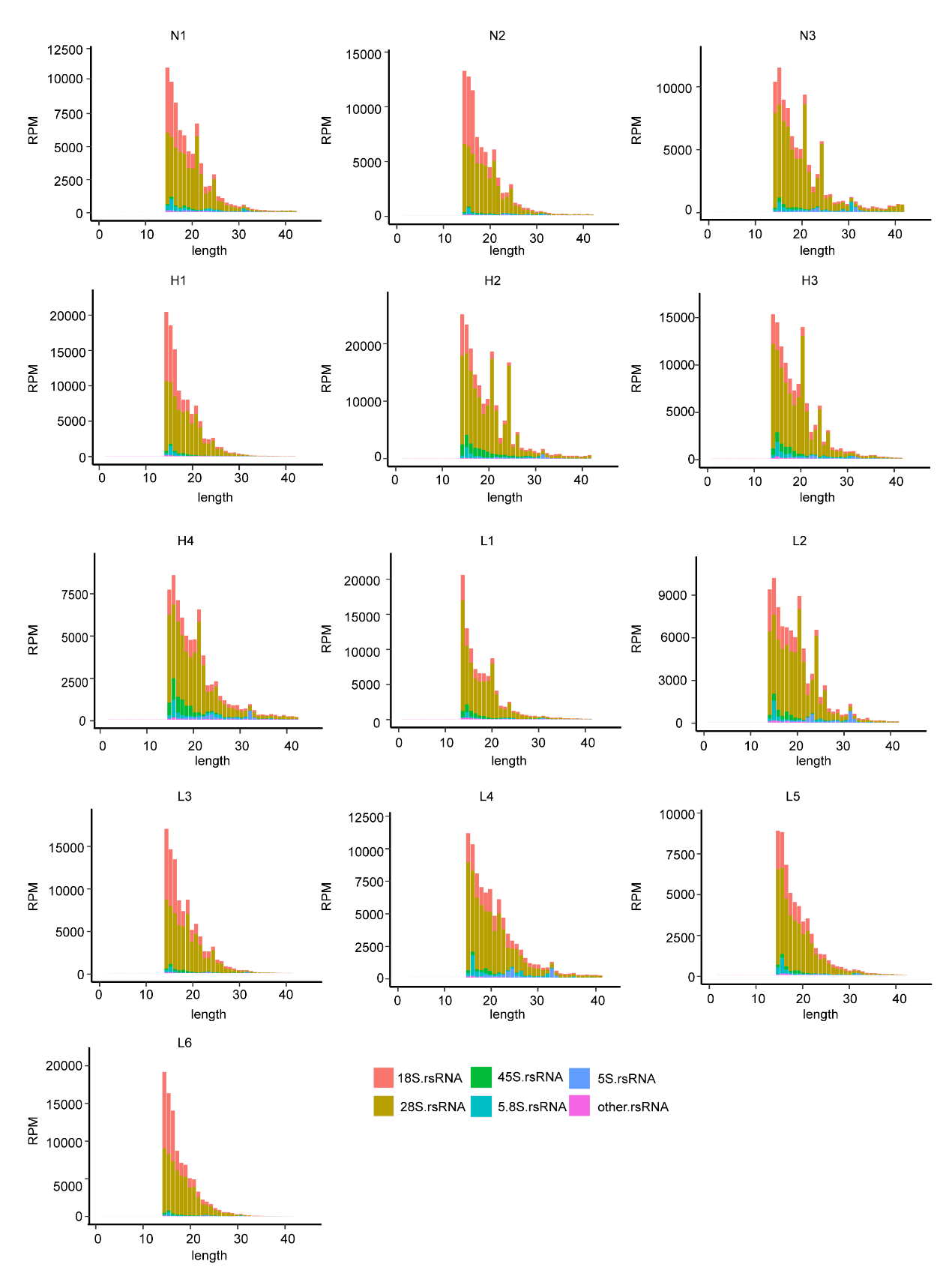
**

**Figure S4:** Mapping of overall length displaying relative rsRNA read distributions in 13 exosome samples in small RNA-seq.

**
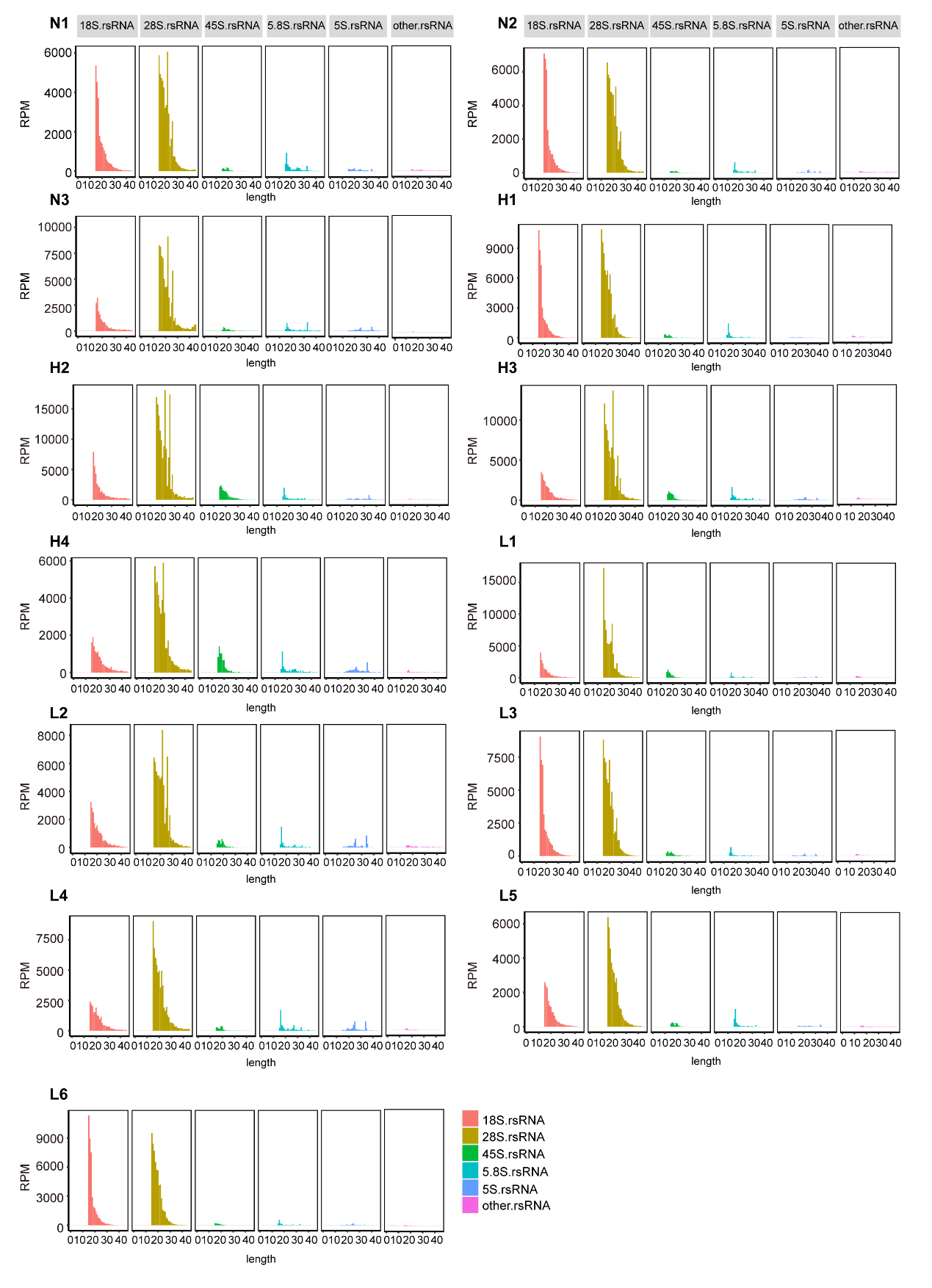
Figure S5:** Results revealing the dynamic rsRNAs landscape measured in discovery cohort.

**
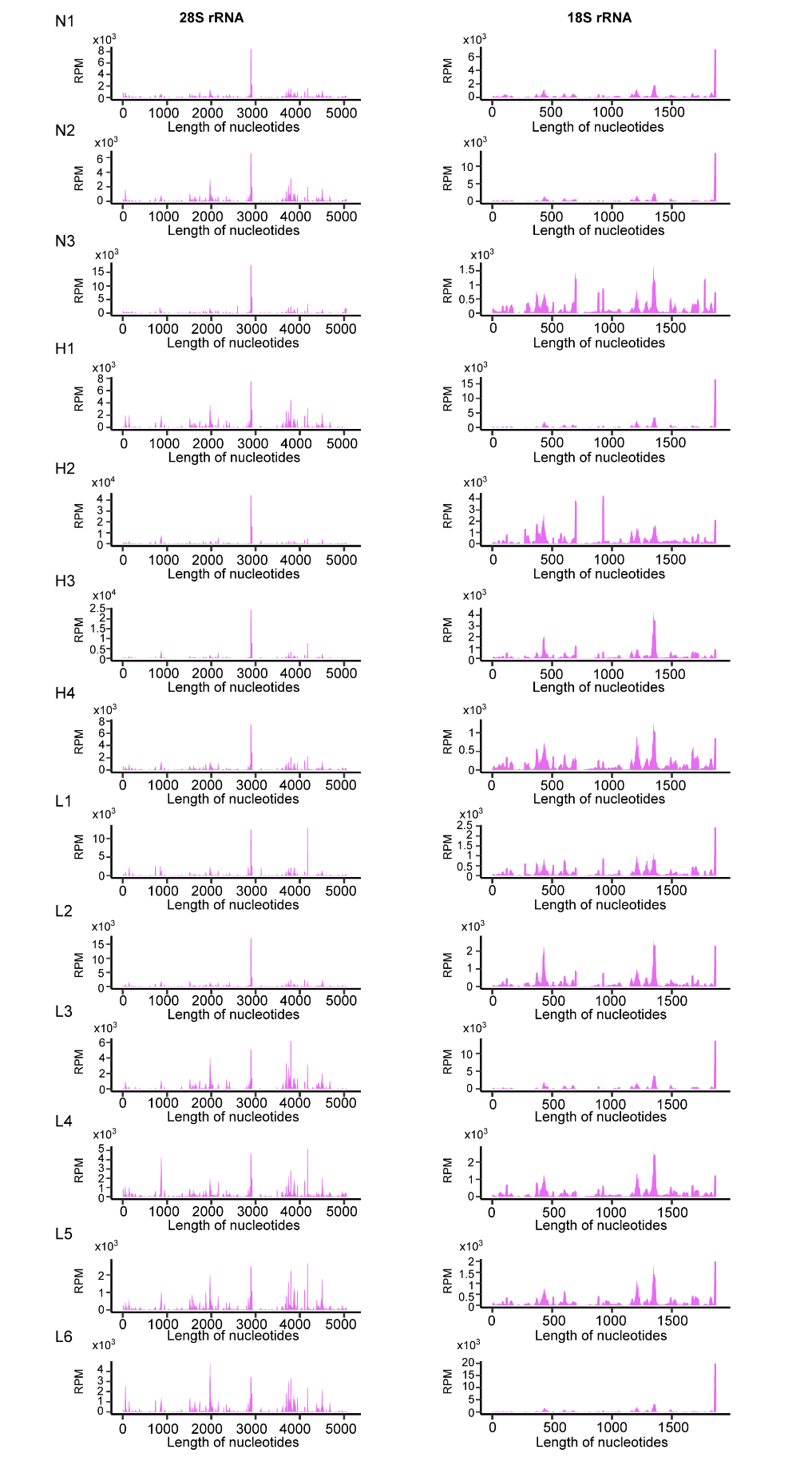
**

**Figure S6:** Results showing the dynamic rsRNAs landscape derived from rRNA-28s and rRNA-18s detected in discovery cohort.

**
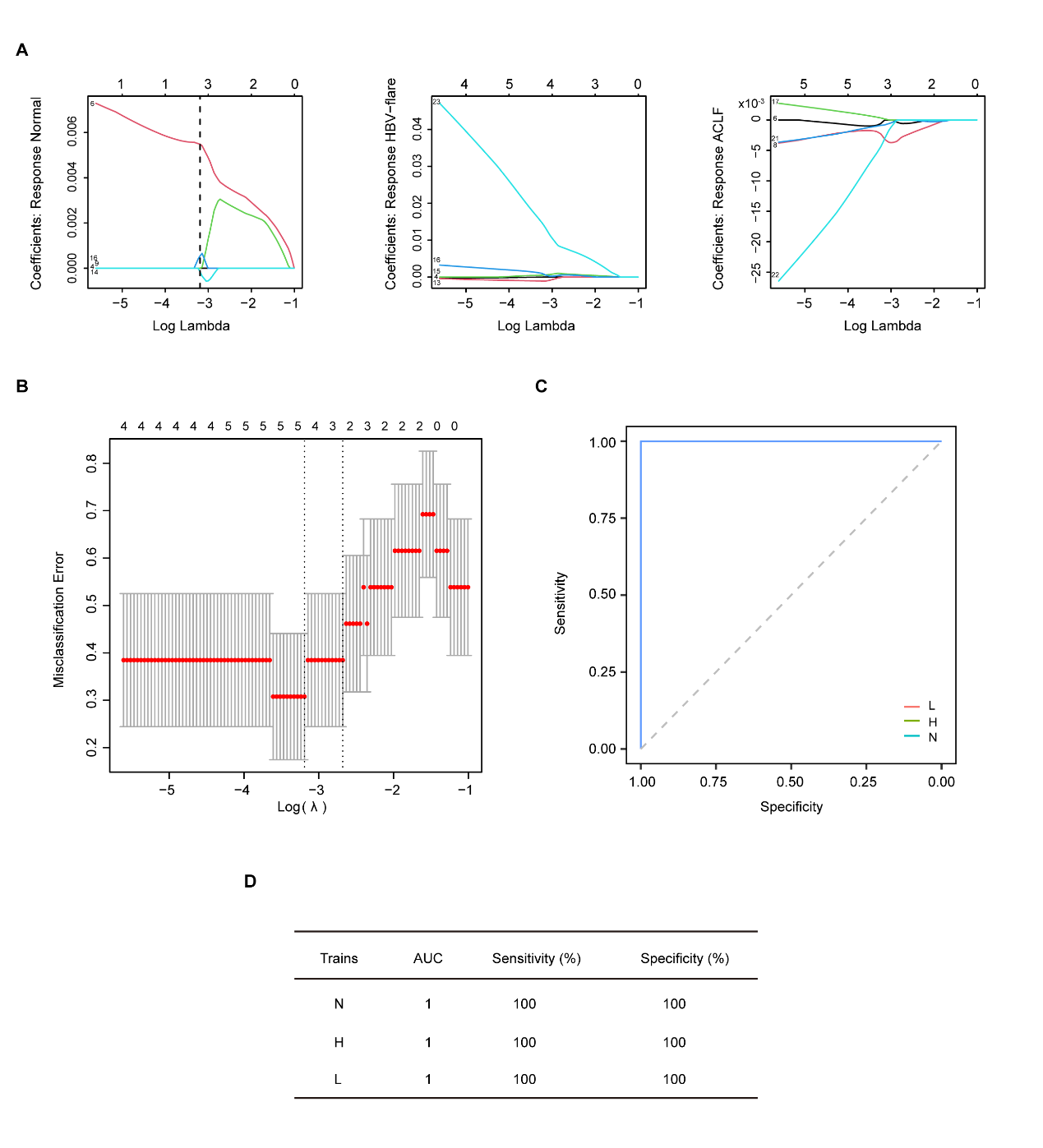
**

**Figure S7:** The construction of a molecule signature model for candidate sncRNAs in the RNA-seq data from the discovery cohort. **(A)** Schematic diagram of LR screening in three different RNA sequencing groups. **(B)** The misclassification error diagram for the RNA-seq model in discovery cohort. **(C)** AUC curve for candidate sncRNAs in the RNA-seq model. **(D)** The Model evaluation for candidate sncRNAs in the RNA-seq model.


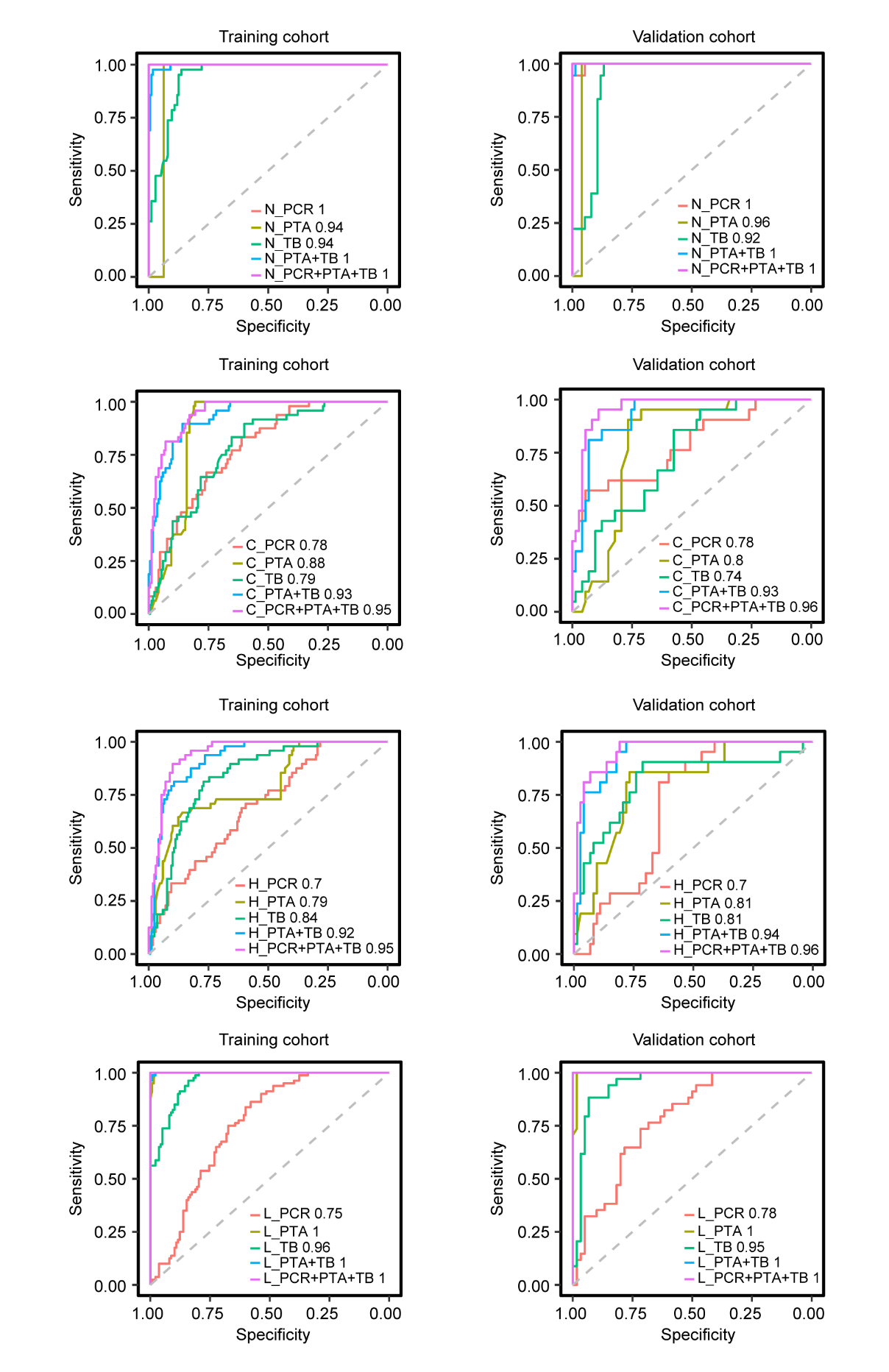


**Figure S8:** Comparison of MTA-RNA signature molecules with current traditional clinical guidelines for TB and PTA, respectively.

**
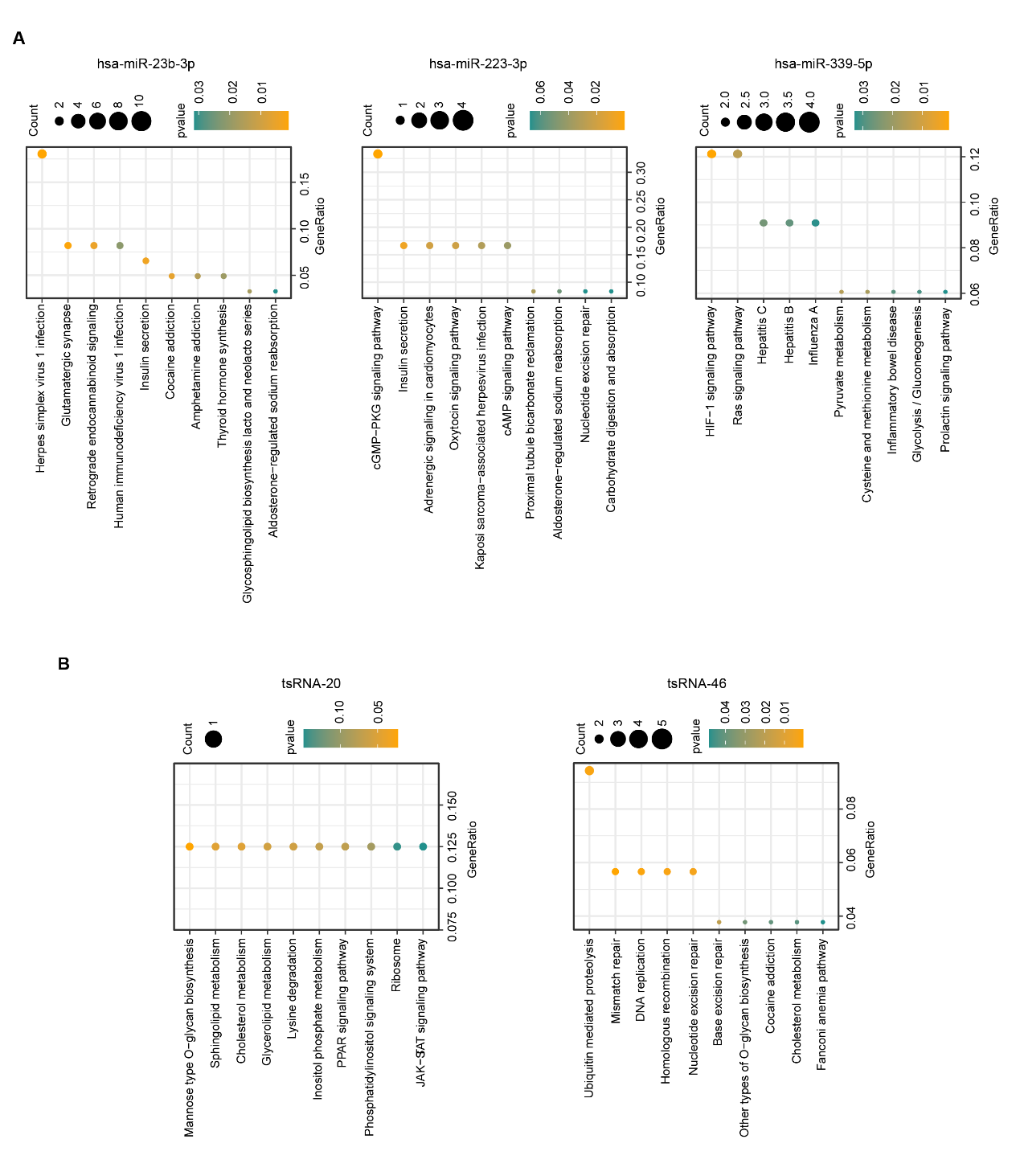
**

**Figure S9:** The KEGG pathway analysis for the MTR-RNA signature including hsa-miR-23b-3p, hsa-miR-223-3p and hsa-miR-339-5p **(A)**, and tsRNA-20 and tsRNA-46 **(B)**.
